# Elevated Cortisol Leaves Working Memory Unaffected in Both Men and Women

**DOI:** 10.3389/fnhum.2018.00299

**Published:** 2018-07-24

**Authors:** Robyn Human, Michelle Henry, W. Jake Jacobs, Kevin G. F. Thomas

**Affiliations:** ^1^ACSENT Laboratory, Department of Psychology, University of Cape Town, Rondebosch, South Africa; ^2^Academic Development Programme, Centre for Higher Education Development, University of Cape Town, Rondebosch, South Africa; ^3^Anxiety Research Group, Department of Psychology, University of Arizona, Tucson, AZ, United States

**Keywords:** cortisol, psychological stress, sex differences, Trier Social Stress Test (TSST), working memory

## Abstract

Activation of the hypothalamic-pituitary-adrenal (HPA) axis (as might occur, for example, when the organism encounters a threat to allostatic balance) leads to the release of cortisol into the bloodstream and, ultimately, to altered neural functioning in particular brain regions (e.g., the prefrontal cortex (PFC)). Although previous studies suggest that exposure to acute psychosocial stress (and hence, presumably, elevation of circulating cortisol levels) enhances male performance on PFC-based working memory (WM) tasks, few studies have adequately investigated female performance on WM tasks under conditions of elevated cortisol. Hence, we compared associations between elevated (relative to baseline) levels of circulating cortisol and *n*-back performance in a South African sample (38 women in the late luteal phase of their menstrual cycle, 38 men). On Day 1, participants completed practice *n*-back tasks. On Day 2, some completed the Trier Social Stress Test (TSST), whereas others experienced a relaxation period, before completing 1-back and 3-back tasks. We measured self-reported anxiety and salivary cortisol at baseline, post-manipulation and end of session. We reconstituted group assignment so that all women with elevated cortisol were in one group (EC-Women; *n* = 17), all men with elevated cortisol were in another (EC-Men; *n* = 19), all women without elevated cortisol were in a third (NoEC-Women; *n* = 21), and all men without elevated cortisol were in a fourth (NoEC-Men; *n* = 19) group. Analyses suggested this reconstitution was effective: in EC, but not NoEC, groups cortisol levels rose significantly from baseline to post-manipulation. Analyses of *n*-back data detected significant relations to task load (i.e., better performance on 1-back than on 3-back tasks), but no significant relations to sex, performance accuracy/speed, or cortisol variation. The data patterns are inconsistent with reports describing sex differences in effects of stress on WM performance. We speculate that cross-study methodological differences account for these inconsistencies, and, particularly, that between-study variation in the magnitude of baseline cortisol levels might affect outcomes. For instance, diurnal cortisol rhythms of South African samples might have flatter curves, and lower baseline values, than predominantly Caucasian samples from the United States and western Europe due to greater prenatal and lifetime stress, more socioeconomic disadvantage and faster ancestral life history (LH) strategies. We describe ways to disconfirm this hypothesis, and urge further cross-national research exploring these possibilities.

## Introduction

The construct of working memory (WM) describes a class of memory produced by a set of neural processes that underpin a variety of higher-order cognitive functions. These processes are integral in coordinating the temporary storage and subsequent manipulation of information that might assist the organism in achieving a variety of goal-directed behaviors, especially when that information must be applied outside of the immediate context (Baddeley and Hitch, 1974; Baddeley, 1986; D’Esposito and Postle, 2015). The purpose of the present investigation was to determine if elevated cortisol levels immediately and differentially affect WM in men and women. This proposal is based on evidence that a chain of predictable neurobiological events follow any threat to allostatic balance (McEwen, 2005). In humans, activation of the hypothalamic-pituitary-adrenal (HPA) axis leads to the release of cortisol into the bloodstream. After crossing the blood-brain barrier, cortisol and related hormones bind to glucocorticoid receptors (GRs), which, although spread throughout the brain, occur in particular abundance in the prefrontal cortex (PFC), hypothalamus and hippocampus (Reul and De Kloet, 1985; Alderson and Novack, 2002; Kemeny, 2003). Once bound, these hormones alter neural functioning in those regions. Of particular interest here is the alteration of PFC functioning when cortisol levels are elevated above baseline, and consequent effects on WM performance (Owen, 1997; Baddeley, 2001; Wolf, 2003; Arnsten, 2009).

Previous studies, built on this chain of evidence, demonstrate that acute psychosocial stress (and consequent elevations in cortisol levels) negatively affect speed and accuracy in the performance of WM tasks, especially at greater cognitive loads (e.g., Lupien et al., 1999; Wolf et al., 2001; Mizoguchi et al., 2004; Elzinga and Roelofs, 2005; Oei et al., 2006; Schoofs et al., 2008, 2009; Luethi et al., 2009; Barsegyan et al., 2010). A smaller group of studies suggest, however, that such cortisol elevations improve, or may have no effect, on WM task performance (Kuhlmann et al., 2005; Oei et al., 2009; Weerda et al., 2010; Stauble et al., 2013). Both groups of studies, however, enrolled only male participants. Although this is a justifiable position given their logic, it does mean these studies leave unexplored the nature of relations between stressor-induced increases in cortisol levels and female performance on WM tasks.

Although under normal circumstances there are negligible sex differences in terms of HPA-axis functioning, men generally exhibit greater stressor-induced increases in cortisol levels than women (Kudielka and Kirschbaum, 2005; Uhart et al., 2006; Kudielka et al., 2009). However, women who are in the late luteal phase of the menstrual cycle and free of oral contraceptives exhibit post-stressor cortisol responses comparable to those of men (Kirschbaum et al., 1999). This fact permits us to compare directly the performance of men and women on tasks representing WM processing in the presence of elevated cortisol.

Under normal circumstances, men and women perform differently on WM-related tasks (Speck et al., 2000; Lynn and Irwing, 2008; but see Evans and Hampson, 2015). These functional differences extend to performance on cognitive tasks related to WM under conditions of elevated cortisol. Although not testing WM directly, several well-designed studies demonstrate that acute exposure to a psychosocial stressor differentially affects performance by men and women on tasks requiring activity in neural regions that support WM processing (Jackson et al., 2006; Preston et al., 2007; Porcelli and Delgado, 2009; van den Bos et al., 2009; Thomas et al., 2010; but see Starcke and Brand, 2016).

Studies featuring exposure to a stressor and consequent elevated cortisol, and focusing on WM, report enhanced male performance but either impaired or unaffected female performance on an *n*-back task (Cornelisse et al., 2011; Schoofs et al., 2013). Cautious interpretation of these data is warranted because Cornelisse et al. (2011) did not control for female use of oral contraceptives or menstrual cycle phase, and, although Schoofs et al. (2013) did use such controls, their findings have yet to be replicated.

In the only other study to specifically investigate cortisol-related sex differences in WM performance, Zandara et al. (2016) found that women whose cortisol levels decreased from pre- to post-manipulation measurement (*n* = 6) showed significantly improved performance, across that period, on a forward digit span test. On a backward digit span task, however, there were neither detectable sex differences nor significant changes in pre- to post-manipulation performance. Although the design did not control for female oral contraceptive use or for menstrual cycle phase, the authors did control for these factors statistically. After doing so, they found no detectable effects on digit span performance. Again, the results should be interpreted cautiously given that: (a) they have yet to be replicated, (b) group sizes with regard to oral contraception/menstrual cycle phase were small (*n* ≤ 12), and (c) there is some debate about the efficacy of forward digit span tasks as a measure of WM, primarily because these tasks do not require manipulation of the presented information and do not include a reaction time component (Jarrold and Towse, 2006; Lynn and Irwing, 2008; Schoofs et al., 2008; Egeland, 2015).

In summary, although previous studies suggest that stress-induced cortisol elevations enhance male performance on PFC-based WM tasks, few studies have adequately investigated female performance on WM tasks under such conditions. Therefore, in the present study, we exposed naturally cycling women in the late luteal phase of the menstrual cycle, and men, to the Trier Social Stress Test (TSST; Kirschbaum et al., 1993). We then compared the performance of women and men with elevated cortisol against that of women and men without elevated cortisol on an *n*-back task, a commonly accepted measure of the WM construct that reliably activates the PFC (Owen et al., 2005; Jaeggi et al., 2010), with some sex-specific differentiation (Speck et al., 2000; Li et al., 2010; although see Kane et al., 2007 and Schmidt et al., 2009 for contrasting data).

An important methodological consideration here is that performance characteristics on *n*-back tasks vary as a function of the level of cortisol and the specific version of the *n*-back being administered. On 0-back or 1-back tasks, for example, elevated cortisol has little or no effect on performance; in contrast, on 2- or 3-back tasks, the presence of elevated cortisol has medium- to large-sized effects on performance, especially in men (Schoofs et al., 2008, 2013; Cornelisse et al., 2011). Hence, we used performance on a 1-back task to contrast with that on a 3-back task.

Based on the methodological considerations regarding relations between level of cortisol and performance on *n*-back tasks, we predicted that: (a) overall WM performance, as measured by the *n*-back, is faster and more accurate on a 1-back than on a 3-back task regardless of biological sex or of exposure to a cortisol-elevating psychosocial stressor. We also sought to replicate the findings that, on a 3-back task, (b) men with cortisol elevations show enhanced performance, whereas (c) women with cortisol elevations show impaired performance (see Cornelisse et al., 2011; Schoofs et al., 2013).

## Materials and Methods

### Design and Setting

This quasi-experimental study took place over two consecutive days, permitting us to ensure that, by the end of Day 1, all participants understood the requirements and nature of the *n*-back before undertaking the Day 2 tasks. On each day, the participant entered the laboratory at 16:00 h or 18:00 h and completed all procedures within 2 h, ensuring control of cortisol’s circadian cycle, maximizing potential for elicitation of a strong HPA-axis response to the stressor and permitting us to investigate the effects of cortisol elevations occurring outside the normal diurnal cycle (Dickerson and Kemeny, 2004; Kudielka et al., 2004, 2009; Maheu et al., 2005).

### Participants

We recruited undergraduate students (45 men and 57 women), between the ages of 18 and 25 years (*M* = 19.33, *SD* = 1.51). Of these, 24 met at least one of the exclusion criteria listed below. Also, one set of cortisol data showed unusual patterns at baseline (>18 *SD* above the mean for participants exposed to the stressor), and another was lost due to experimenter error (see Table 1). Hence, our final sample consisted of data obtained from 38 men and 38 women. Each received course credit in exchange for participation.

**Table 1:** Reasons recruited participants were excluded from final data analysis (*n* = 26).

	Number excluded	
Reason for exclusion	Men	Women
BDI-II score ≥ 29	3	3
Began menstruation on Day 2 of the study	N/A	3
Did not arrive for Day 2	2	3
Accepted ethical right to withdraw from the study	0	1
Did not meet criterion on Day 1 *n*-back practice trials	2	0
More than 1 day out of late luteal phase during Day 2	N/A	7
Unusual baseline cortisol level	0	1
Cortisol data lost	0	1
Total excluded	7	19

*Note. BDI-II, Beck Depression Inventory—Second Edition*.

#### Exclusion Criteria

We asked those people using any form of steroid medication, and women using any form of oral contraceptive, not to apply to the study. We used this exclusion criterion because these medications affect the magnitude of cortisol response to psychosocial stressors. We also asked those women experiencing an irregular menstrual cycle not to apply. We permitted women reporting a regular menstrual cycle to enrol because the study design specified testing women during the late luteal phase of the menstrual cycle (i.e., the 6-day window preceding the start of menses; Ferin et al., 1993; Symonds et al., 2004). Each woman indicated the date she expected to begin her next period and received an appointment within the 6 days preceding that date. Due to within- and between-woman variability in overall menstrual cycle length, this method appears to be an accurate way to predict phase of the menstrual cycle (Sherman and Korenman, 1975; Cole et al., 2009). Each enrolled woman contacted the experimenter on the first day of her next period to confirm, post-experimentally, the phase of the menstrual cycle during which she had been tested.

We excluded enrolled participants who: (a) scored 29 or above on the Beck Depression Inventory—Second Edition (BDI-II; Beck et al., 1996); (b) self-reported beginning menstruation on either Day 1 or Day 2 of the experimental protocol; (c) did not arrive for the Day 2 session; or (d) withdrew from the study at any point during the Day 1 or Day 2 protocols. We also excluded data sets from individuals who did not meet the *n*-back performance criterion during the Day 1 practice session (see below), and from women who reported, after completing the experimental protocols, they were more than 1 day outside of the late luteal phase of the menstrual cycle during Day 2.

This study was carried out in accordance with the recommendations of the University of Cape Town’s Guidelines for Human Research, as interpreted by the Research Ethics Committees of that institution’s Faculty of Health Sciences and Department of Psychology, with written informed consent from all subjects. All subjects gave written informed consent in accordance with the Declaration of Helsinki (World Medical Association, 2013). The protocol was approved by the Research Ethics Committees of the University of Cape Town’s Faculty of Health Sciences and Department of Psychology.

### Materials and Procedures

#### Day 1

A female research assistant (RA) met participants at the laboratory. She assigned each participant pseudo-randomly to one of four groups: TSST-Women, TSST-Men, Relax-Women, and Relax-Men. Each participant then read and signed a consent form and completed the BDI-II and Form Y-2 of the State-Trait Anxiety Inventory (STAI; Spielberger et al., 1983). The four men and four women who met the BDI-II exclusion criterion did not self-report levels of depression requiring immediate intervention. Hence, the RA provided counselling referrals and dismissed them from the study. Results from the STAI-Trait form served as a measure of general anxiety levels, permitting us to ensure there were no detectable between-group differences in everyday experiences of anxiety.

The remaining participants then completed a set of practice *n*-back tasks. They completed one block of 20 0-back trials, followed by one block of 20 1-back trials, followed by one block of 20 3-back trials.

A standard desktop computer presented the *n*-back tasks using E-prime software version 1.1 (Psychology Software Tools, Pittsburgh, PA, USA). The E-prime *n*-back script was modified from one similar to that at https://step.talkbank.org/scripts-plus/. Participants saw a random series of letters, presented one at a time, on a computer screen. They were instructed to determine if the presented letter was a “target” or a “non-target.” If the former, they pressed the *F* key on the keyboard; if the latter, they pressed the *J* key. Each letter was displayed on-screen for 500 ms with an inter-stimulus interval of 3518 ms. Participants were required to achieve an accuracy score of at least 70% on the 0-back before proceeding to the 1-back, 70% on the 1-back before proceeding to the 3-back, and 70% on the 3-back to end the practice tasks.

At the end of the Day 1 session, the RA reminded participants of the appointment scheduled for the next day, asked them not to smoke, consume any food or drink, chew gum, or engage in physical exercise for 2 h before the start of the Day 2 session. This reminder paralleled protocols described by others (e.g., Kirschbaum et al., 1993; Schoofs et al., 2008; Verdejo-Garcia et al., 2015).

#### Day 2

**Figure 1:**
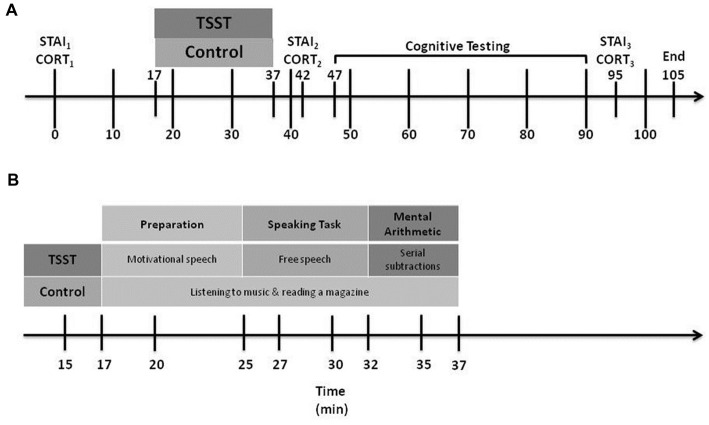
Day 2 experimental procedures. CORT, cortisol levels; STAI, State-Trait Anxiety Inventory; TSST, Trier Social Stress Test. Part **(A)** shows the overall procedure, while Part **(B)** shows details of the experimental manipulation.

The same RA met returning participants at the laboratory, and reminded them of their ethical right to withdraw from the study at any time without penalty. Figure 1 illustrates the timeline of Day 2 experimental events.

Participants rated their current level of anxiety thrice using the STAI-State form: the first, at baseline, shortly after entering the laboratory (STAI_B_), the second at 5 min following the end of the stress or control manipulation (STAI_1_) and the third at 45 min after the end of the manipulation (STAI_2_).

The RA collected three saliva samples using SARSTEDT Salivette^®^ Cortisol swabs (Sarstedt, Nümbrecht, Germany): the first, at baseline, shortly after the participant entered the laboratory (CORT_B_), the second at 5 min after the stress or control manipulation ended (CORT_1_) and the third at 45 min after the manipulation ended (CORT_2_). Immediately after each collection, the RA stored the saliva samples in individual, labeled tubes and placed them in a freezer where they were stored at −20°C. Upon completion of data collection, we transported the tubes to a laboratory for analysis. Samples were analyzed using a competitive electrochemiluminescent immunoassay on the Roche Cobas 6000 (Roche Diagnostics GmbH, Mannheim, Germany) with a coefficient of variation of 4%.

At the end of the session, the RA debriefed participants completely and reminded the women to contact the experimenter on the first day of their next period. The study was then concluded.

##### Experimental Manipulation

Participants in the TSST-Women and TSST-Men groups completed a modified form of the TSST to induce cortisol release. The TSST, which involves public speaking and mental arithmetic tasks, on the average induces large increases in cortisol levels (Kirschbaum et al., 1993; Dickerson and Kemeny, 2004; Foley and Kirschbaum, 2010). In the current study, participants were told that an interviewing panel (one man and one woman) in a separate room would analyze their speech and behavior with the help of a video camera. Participants were then instructed to write and present a speech detailing their suitability for a job of their choosing. After 10 min of preparation, the RA escorted the participant to a room illuminated by a harsh, bright light. There, the participant spoke extemporaneously for 5 min while being observed by the interviewing panel. From this point, the protocol (including the actual speech and the arithmetic task) followed that described by Kirschbaum et al. (1993) closely.

Administration of the control procedures occurred in the same room as those of the stress induction. In this case, however, the room had normal lighting, no video camera and no judges. Participants in the Relax-Women and Relax-Men groups sat in comfortable chairs, read neutral-content magazines and listened to relaxing music for 20 min.

##### The *n*-Back Tasks

Following the experimental manipulation, participants completed a 0-back task (1 block, 24 trials). They then completed four alternating 1-back and 3-back blocks (24 trials each). On eight trials within each block, the correct response was *F* (“target”); on the remaining 16, the correct response was *J* (“non-target”). Following Schoofs et al. (2008, 2013), the first three trials of each block were non-targets, and we did not include their data in the final analysis. This preparation permitted us to examine performance on three *n*-back versions (0-, 1- and 3-back) while the participant was under the influence of elevated cortisol (see above and Sliwinski et al., 2006, for arguments detailing the value of this approach).

### Statistical Analysis

After applying the exclusion criteria described above, the groups were constituted as follows: TSST-Women (*n* = 20), TSST-Men (*n* = 17), Relax-Women (*n* = 18), and Relax-Men (*n* = 21). Based on the moderate effect sizes reported in studies describing relations between stress and working memory performance (e.g., Schoofs et al., 2013), a power analysis indicated that an *N* of 76 is sufficient to detect the effects under consideration. Furthermore, our sample size compares favorably to those used in similar studies (Schoofs et al., 2008, 2013; Cornelisse et al., 2011).

We analyzed the data using SPSS (version 23.0), and set the Type I error rate at 0.05 unless otherwise specified. In cases where assumptions of parametric statistical tests were violated, we made suitable adjustments (e.g., log transformations of data).

## Results

Common findings in this area of research are that: (a) there is considerable variability in response to laboratory-based stress induction methods (e.g., Buchanan and Tranel, 2008; van den Bos et al., 2009; Schlotz et al., 2011), and (b) more men than women show increased cortisol after TSST exposure (e.g., Stroud et al., 2002; Elzinga and Roelofs, 2005). These patterns were present in our data: post-manipulation measurement detected elevated cortisol levels in 12 of 20 women (60%) and 14 of 17 men (82%), in the TSST condition. In the Relax condition, 5 of 18 women (28%) and 5 of 21 men (24%), showed elevated post-manipulation cortisol levels.

Because our questions focused on relations between elevated cortisol and WM performance, we reconstituted the group assignment so that women with elevated cortisol (EC) were in one group (EC-Women; *n* = 17), men with elevated cortisol were in another (EC-Men; *n* = 19), women without elevated cortisol were in a third (NoEC-Women; *n* = 21) and men without elevated cortisol were in a fourth (NoEC-Men; *n* = 19). Here, we defined “elevated cortisol” as any increase in cortisol level from CORT_B_ to CORT_1_ (i.e., any elevation from baseline to the immediate post-manipulation measurement point). Subsequent analyses compared self-report and physiological measures of stress, and working memory performance, across the EC-Women, EC-Men, NoEC-Women and NoEC-Men groups.

### Sample Characteristics

A series of one-way ANOVAs detected no statistically significant between-group differences in mean age, BDI-II scores, or STAI-Trait scores (see Table 2).

**Table 2:** Between-group comparisons: participant age and self-reported depression and anxiety (*N* = 76).

	Group						
Measure	EC-Women (*n* = 17)	EC-Men (*n* = 19)	NoEC-Women (*n* = 21)	NoEC-Men (*n* = 19)	*F*	*p*	ESE
Age	19.12 (1.11)	19.84 (1.89)	18.95 (1.16)	18.89 (0.99)	2.05	0.12	0.08
BDI-II	13.06 (6.55)	9.89 (6.13)	12.43 (6.18)	10.42 (5.31)	1.19	0.32	0.05
STAI-Trait	40.94 (9.15)	37.37 (5.98)	43.67 (10.97)	39.89 (9.64)	1.61	0.19	0.06

*Note. Data presented are means with standard deviations in parentheses. ESE, effect size estimate (in this case, β^2^); BDI-II, Beck Depression Inventory-Second Edition; STAI, State-Trait Anxiety Inventory*.

### Group Assignment Check

We conducted 4 × 3 (Group [EC-Women, EC-Men, NoEC-Women, NoEC-Men] × Time [Baseline, Time 1, Time 2]) repeated-measures ANOVAs on data for the STAI-State scores and salivary cortisol levels (see Table 3 for descriptive statistics). Planned comparisons tested *a priori* hypotheses regarding between- and within-group differences.

**Table 3:** Descriptive statistics: self-report and physiological measures (*N* = 76).

	Group			
Measure	EC-Women (*n* = 17)	EC-Men (*n* = 19)	NoEC-Women (*n* = 21)	NoEC-Men (*n* = 19)
STAI-State				
STAI_B_	36.94 (8.50)	34.58 (6.69)	37.43 (10.97)	34.58 (11.78)
STAI_1_	45.53 (11.73)	39.16 (12.31)	36.43 (11.60)	30.68 (12.43)
STAI_2_	32.94 (7.20)	29.26 (6.83)	33.00 (8.33)	31.05 (6.56)
Cortisol				
CORT_B_	1.08 (1.06)	1.96 (2.04)	1.97 (2.28)	2.57 (2.86)
CORT_1_	4.11 (2.89)	7.03 (4.65)	1.55 (1.90)	1.82 (2.23)
CORT_2_	1.30 (0.81)	2.65 (2.35)	1.16 (1.37)	1.55 (1.97)

*Note. Data presented are means with standard deviations in parentheses. STAI, State-Trait Anxiety Inventory. Cortisol levels are measured in nanomoles per liter (nmol/l). Where cortisol levels for a participant were measured at <0.50 nmol/l, 0.45 nmol/l was used as an estimate*.

#### Subjective Anxiety

The analysis detected a significant main effect of Time, *F*_(2,144)_ = 16.18, *p* < 0.001, $\eta_{\text{p}}^{2}$ = 0.18, but not of Group, *F*_(3,72)_ = 2.11, *p* = 0.11, $\eta_{\text{p}}^{2}$ = 0.08. The analysis also detected a significant Group × Time interaction, *F*_(6,144)_ = 4.12, *p* = 0.001, $\eta_{\text{p}}^{2}$ = 0.15.

We performed planned contrasts on the Time and Group × Time effects. Regarding the main effect of Time, the STAI_B_ vs. STAI_1_ contrast was not significant, *t*_(133.39)_ = −1.01, *p* = 0.31, Cohen’s *d* = 0.17, but the STAI_B_ vs. STAI_2_ and STAI_1_ vs. STAI_2_ contrasts were significant at the Bonferroni-corrected *p* of 0.017, *t*_(144.49)_ = 3.27, *p* = 0.001, *d* = 0.53 and *t*_(118.77)_ = 3.62, *p* < 0.001, *d* = 0.59, respectively.

Regarding the Group × Time interaction, two of the five contrasts were significant at the Bonferroni-corrected *p* of 0.01. The first compared change from STAI_B_ to STAI_1_ in the EC-Women and EC-Men groups, taken together, *t*_(216)_ = −2.89, *p* = 0.004, *d* = 0.64. The second compared, at STAI_1_, the average STAI-State scores of the EC-Women and EC-Men groups, taken together, vs. those of the NoEC-Women and NoEC-Men groups, taken together, *t*_(216)_ = 3.96, *p* < 0.001, *d* = 0.70. The other three contrasts (first, comparing change from STAI_B_ to STAI_1_in the NoEC-Women and NoEC-Men groups, taken together; second, comparing, at STAI_B_, the scores of the EC-Women and EC-Men groups, taken together, vs. those of the NoEC-Women and NoEC-Men groups, taken together; and third, comparing, at STAI_2_, the scores of the EC-Women and EC-Men groups, taken together, vs. those of the NoEC-Women and NoEC-Men groups, taken together) were not significant, *t*_S_ < 1.13, *p*_S_ > 0.26, *d*_S_ < 0.27. This set of analyses confirms that, while undergoing cognitive testing, participants in the EC and NoEC groups were experiencing different levels of self-reported anxiety.

#### Cortisol Levels

**Figure 2:**
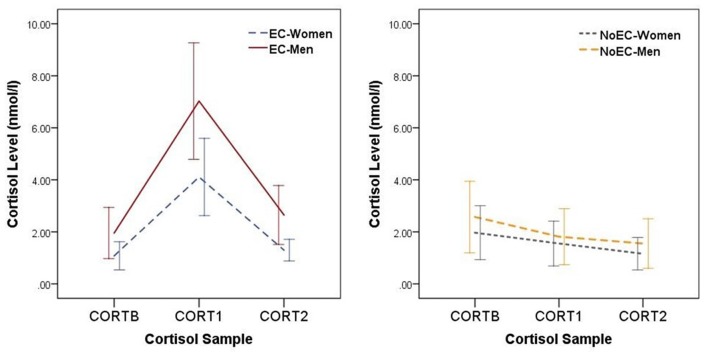
Changes in cortisol levels across the Day 2 experimental procedures. Error bars represent standard deviations. Data for elevated cortisol (EC) groups are presented separately from those for NoEC groups to allow easier viewing of the magnitude of error bars. CORT_B_, cortisol at baseline; CORT_1_, cortisol at the first post-manipulation measurement point (i.e., 5 min post-manipulation); CORT_2_, cortisol at the second post-manipulation measurement point (i.e., 45 min post-manipulation).

Figure 2 shows the fluctuations in group cortisol levels across the Day 2 experimental procedures.

Mauchly’s test detected a violation of the assumption of sphericity, $\chi_{\left(2\right)}^{2}$ = 8.43, *p* = 0.02. Hence, we corrected degrees of freedom using Huynh-Feldt estimates of sphericity, *ε* = 0.96. The analysis detected main effects of Time, *F*_(1.92,138.16)_ = 38.20, *p* < 0.001, $\eta_{\text{p}}^{2}$ = 0.35, and of Group, *F*_(3,72)_ = 4.75, *p* = 0.004, $\eta_{\text{p}}^{2}$ = 0.17, and a Group × Time interaction, *F*_(5.76,138.16)_ = 17.64, *p* < 0.001, $\eta_{\text{p}}^{2}$ = 0.43.

We performed planned contrasts on all three effects. Regarding the main effect of Time, the CORT_B_ vs. CORT_1_ and CORT_1_ vs. CORT_2_ contrasts were significant at the Bonferroni-corrected *p* of 0.017, *t*_(121.03)_ = −3.28, *p* = 0.001, *d* = 0.54, and *t*_(107.80)_ = 3.97, *p* < 0.001, *d* = 0.65, respectively, but the CORT_B_ vs. CORT_2_ contrast was not, *t*_(144.41)_ = 0.79, *p* = 0.43, *d* = 0.13. This set of analyses confirms an impression given by Figure 2: Cortisol levels at baseline were equivalent to those at 45-min post-manipulation, with levels at 5-min post-manipulation greater than those at both baseline and 45-min post-manipulation.

Regarding the main effect of Group, two contrasts (EC-Women vs. EC-Men, and the EC-Women and EC-Men groups taken together vs. the NoEC-Women and NoEC-Men groups taken together) were significant at the Bonferroni-corrected *p* of 0.017, *t*_(91.86)_ = −2.83, *p* = 0.006, *d* = 0.56, and *t*_(161.22)_ = 3.45, *p* < 0.001, *d* = 0.48, respectively, while the NoEC-Women vs. NoEC-Men contrast was not, *t*_(106.63)_ = −1.07, *p* = 0.29, *d* = 0.20. This set of analyses confirms that our reconstituted group assignment effectively separated those with elevated cortisol from those without.

Regarding the Group × Time interaction effect, two of the five contrasts were significant at the Bonferroni-corrected *p* of 0.01. The first of these compared change from CORT_B_ to CORT_1_ in the EC-Women and EC-Men groups, taken together, *t*_(40.70)_ = −5.86, *p* < 0.001, *d* = 1.32. The second compared, at CORT_1_, the average cortisol levels of the EC-Women and EC-Men groups, taken together, vs. those of the NoEC-Women and NoEC-Men groups, taken together, *t*_(46.09)_ = 1.57, *p* < 0.001, *d* = 1.25. The other three contrasts (first, comparing change from CORT_B_ to CORT_1_in the NoEC-Women and NoEC-Men groups, taken together; second, comparing, at CORT_B_, the scores of the EC-Women and EC-Men groups, taken together, vs. those of the NoEC-Women and NoEC-Men groups, taken together; and third, comparing, at CORT_2_, the scores of the EC-Women and EC-Men groups, taken together, vs. those of the NoEC-Women and NoEC-Men groups, taken together) were not significant, *t*_S_ < 1.57, *p*_S_ > 0.12, *d*_S_ < 0.38. This set of analyses confirms that, while undergoing cognitive testing, participants in the EC and NoEC groups were experiencing different levels of circulating cortisol.

#### Interim Summary

Analyses of the STAI-State and salivary cortisol data detected: (a) no differences between the EC and the NoEC groups at baseline or at the end-of-session measurement point, (b) differences between those groups at the immediate post-manipulation measurement point, and (c) an increase in self-reported anxiety and physiological stress markers from baseline to the immediate post-manipulation measurement in the EC, but not in the NoEC, groups.

### Working Memory Tasks: Primary Analyses

#### Day 1

Because performance on the Day 1 *n*-back tasks ensured that participants understood the requirements of those tasks, we examined descriptive statistics to give an indication of participants’ performance, and then conducted a repeated-measures ANOVA to investigate whether: (a) participants required more blocks to reach criterion performance as the WM task load increased, and (b) there were between-group differences in number of 0-back, 1-back and 3-back blocks required to reach criterion performance.

For the 0-back task, 73 participants (96%) achieved the required 70% accuracy at their first attempt, and the rest achieved it at the second attempt. For the 1-back task, 66 participants (87%) achieved the required accuracy at their first attempt, and the rest (bar one, who achieved it at the third attempt) achieved it at the second attempt. For the 3-back task, 58 participants (76%) achieved the required accuracy at their first attempt, and most others (14 of 18) achieved it at the second attempt (see Table 4).

**Table 4:** Descriptive statistics: Day 1 *n*-back trials to criterion (*N* = 76).

	Group			
Task load/Trial number	EC-Women (*n* = 17)	EC-Men (*n* = 19)	NoEC-Women (*n* = 21)	NoEC-Men (*n* = 19)
0-back				
One	16	18	21	18
Two	1	1	0	1
1-back				
One	14	18	19	15
Two	2	1	2	4
Three	1	0	0	0
3-back				
One	11	17	17	13
Two	5	2	2	5
Three	1	0	1	1
Four	0	0	1	0

*Note. Data presented indicate number of people who reached criterion on the indicated trial*.

Mauchly’s test detected a violation of the assumption of sphericity, $\chi_{\left(2\right)}^{2}$ = 13.70, *p* = 0.001. Hence, we corrected degrees of freedom using Huynh-Feldt estimates of sphericity, *ε* = 0.91. The analysis detected a main effect of Task Load, *F*_(1.81, 130.39)_ = 7.54, *p* < 0.01, $\eta_{\text{p}}^{2}$ = 0.10, but no main effect of Group, *F*_(3,72)_ = 1.42, *p* = 0.24, $\eta_{\text{p}}^{2}$ = 0.06, and no Group × Task Load interaction, *F*_(5.43,130.39)_ = 0.58, *p* = 0.730, $\eta_{\text{p}}^{2}$ = 0.02.

Regarding the main effect of Task Load, the 0-back vs. 1-back and 1-back vs. 3-back contrasts were not significant at the Bonferroni-corrected *p* of 0.017, *t*_(110.62)_ = −2.10, *p* = 0.038, and *t*_(127.36)_ = −1.90, *p* = 0.06, respectively. However, the 0-back vs. 3-back contrast was significant, *t*_(90.25)_ = −3.57, *p* = 0.001.

#### Day 2

We derived two outcome variables for each block of the 1-back and 3-back tasks: Correctly Identified Stimuli (CIS; the percent of target and non-target letters each participant correctly identified) and Mean Reaction Time (MRT) to CIS. Hence, the data set comprised eight CIS variables and eight MRT variables for each participant (see Table 5).

**Table 5:** Descriptive statistics: Day 2 *n*-back performance (*N* = 76).

		Group			
	Measure	EC-Women (*n* = 17)	EC-Men (*n* = 19)	NoEC-Women (*n* = 21)	NoEC-Men (*n* = 19)
Block 1					
CIS	1-back	95.24 (6.94)	95.74 (6.53)	94.79 (5.61)	96.49 (3.49)
	3-back	87.50 (9.97)^a^	83.21 (8.15)	82.31 (14.92)	87.47 (8.58)
MRT	1-back	581.74 (130.20)	681.72 (203.05)	560.83 (175.51)	606.28 (155.64)
	3-back	799.33 (239.58)^a^	912.45 (359.02)	791.00 (284.22)	853.54 (839.38)
Block 2					
CIS	1-back	93.00 (7.36)	94.74 (4.73)	95.01 (6.30)	92.98 (15.90)
	3-back	82.63 (10.10)	79.95 (13.05)	83.67 (11.23)	79.70 (14.10)
MRT	1-back	621.57 (152.20)	704.01 (212.36)	593.26 (173.14)	585.08 (134.99)
	3-back	848.25 (249.75)	900.66 (382.67)	807.83 (265.17)	916.83 (300.42)
Block 3					
CIS	1-back	95.24 (4.76)	93.99 (7.91)	94.79 (6.55)	92.48 (7.33)
	3-back	88.24 (12.04)	84.96 (11.91)	82.31 (12.79)	86.47 (9.43)
MRT	1-back	619.26 (140.62)	659.08 (206.56)	602.37 (193.02)	643.44 (183.56)
	3-back	767.63 (225.19)	772.31 (330.40)	775.22 (317.99)	835.55 (295.45)
Block 4					
CIS	1-back	93.48 (6.02)	94.24 (7.87)	92.52 (9.59)	93.74 (11.88)
	3-back	84.59 (9.45)	86.72 (11.10)	78.47 (19.66)	79.45 (14.38)
MRT	1-back	598.36 (97.97)	678.71 (236.25)	573.72 (156.81)	620.00 (170.51)
	3-back	802.66 (220.58)	807.55 (325.61)	748.32 (198.36)	878.23 (317.77)

*Note. Data presented are means, with standard deviations in parentheses. CIS, correctly identified stimuli; MRT, mean reaction time (measured in milliseconds). ^a^Data based on *n* = 16 participants. One participant in this group used an incorrect key to provide responses for all non-target trials in this block*.

##### CIS

We conducted a 4 × 2 × 4 (Group [EC-Women, EC-Men, NoEC-Women, NoEC-Men] × Task Load [1-back, 3-back] × Block [1, 2, 3, 4]) repeated-measures ANOVA on this set of data. Because the data were not normally distributed, we log-transformed them and analyzed the transformed set.

Mauchly’s test detected a violation of the assumption of sphericity for the Block factor, $\chi_{\left(5\right)}^{2}$ = 18.58, *p* = 0.002, and the Task Load × Block interaction, $\chi_{\left(5\right)}^{2}$ = 17.26, *p* = 0.004 data. Hence, in both cases we corrected degrees of freedom using Huynh-Feldt estimates of sphericity, *ε* = 0.94. Subsequently, the analysis detected a main effect of Task Load, *F*_(1,71)_ = 85.61, *p* < 0.001, $\eta_{\text{p}}^{2}$ = 0.55, and of Block, *F*_(2.83, 200.64)_ = 3.72, *p* = 0.014, $\eta_{\text{p}}^{2}$ = 0.05. Regarding the main effect of Task Load, participants obtained a greater overall percentage of correct responses on the four 1-back blocks (*M* ± *SE*: 94.30 ± 0.68) than on the four 3-back blocks (*M* ± *SE*: 83.39 ± 1.12). In fact, participants in all four groups appeared to perform equivalently, and almost at ceiling, on the 1-back trials. Regarding the main effect of Block, participants performed best on the first block of trials, and worst on the second and fourth blocks (*M* ± *SE*: block 1 = 90.53 ± 0.77; block 2 = 87.86 ± 1.06; block 3 = 89.72 ± 0.92; block 4 = 87.93 ± 1.10). *Post-hoc* pairwise comparisons, using Tukey’s LSD procedure, detected differences between performance on the first and second blocks, *p* = 0.014, *d* = 0.22, first and fourth blocks, *p* = 0.011, *d* = 0.22, and second and third blocks, *p* = 0.045, *d* = 0.02.

The analysis detected no main effect of Group, *F*_(3,71)_ = 0.48, *p* = 0.695, $\eta_{\text{p}}^{2}$ = 0.02, and no interactions, *F*_S_ < 1.83, *p*_S_ > 0.12, $\eta_{\text{pS}}^{2}$ < 0.06. Hence, neither sex nor variation in cortisol detectably affected the accuracy of *n*-back performance. A series of bivariate correlational analyses, documenting associations between magnitude of cortisol change from Cort_B_ to Cort_1_ and the CIS variables, confirmed the veracity of this fact, *r*_S_ < 0.14, *p*_S_ > 0.25.

##### MRT

As an initial analytic step, we conducted a 4 × 2 × 4 (Group (EC-Women, EC Men, NoEC-Women, NoEC-Men) × Task Load [1-back, 3-back] × Block [1, 2, 3, 4]) repeated-measures ANOVA on this set of data. Because the data were not normally distributed, we analyzed a log-transformed set.

Mauchly’s test detected a violation of the assumption of sphericity for data related to the Block factor, $\chi_{\left(5\right)}^{2}$ = 11.80, *p* = 0.038. Hence, for that factor we corrected degrees of freedom using Huynh-Feldt estimates of sphericity, *ε* = 0.97. The analysis detected a main effect of Task Load, *F*_(1,71)_ = 115.60, *p* < 0.001, $\eta_{\text{p}}^{2}$ = 0.62, and Block, *F*_(2.91,206.83)_ = 3.31, *p* = 0.022, $\eta_{\text{p}}^{2}$ = 0.04, but not Group, *F*_(3,71)_ = 0.51, *p* = 0.679, $\eta_{\text{p}}^{2}$ = 0.02.

Regarding the main effect of Task Load, participants’ reaction time on the four 3-back blocks (*M* ± *SE*: 824.69 ± 31.70) was significantly slower than that on the four 1-back blocks (*M* ± *SE*: 619.89 ± 18.93). Regarding the main effect of Block, participants performed best on the third block of trials and worst on the second block (*M* ± *SE*: block 1 = 723.43 ± 26.26; block 2 = 745.21 ± 25.84; block 3 = 708.45 ± 25.36; block 4 = 712.86 ± 23.70). *Post-hoc* pairwise comparisons, using Tukey’s LSD procedure, detected differences between performance on the first and second blocks, *p* = 0.045, *d* = 0.08, second and third blocks, *p* = 0.002, *d* = 0.14, and second and fourth blocks, *p* = 0.008, *d* = 0.13.

The analysis did not detect interactions between Group and Task Load, *F*_(3,71)_ = 1.39, *p* = 0.254, $\eta_{\text{p}}^{2}$ = 0.06, between Group and Block, *F*_(8.74,206.83)_ = 1.76, *p* = 0.080, $\eta_{\text{p}}^{2}$ = 0.07, or between Group, Task Load, and Block, *F*_(9,213)_ = 0.92, *p* = 0.512, $\eta_{\text{p}}^{2}$ = 0.04. It did, however, detect a Task Load × Block interaction, *F*_(3,213)_ = 0.091, *p* < 0.001, $\eta_{\text{p}}^{2}$ = 0.10.

**Figure 3:**
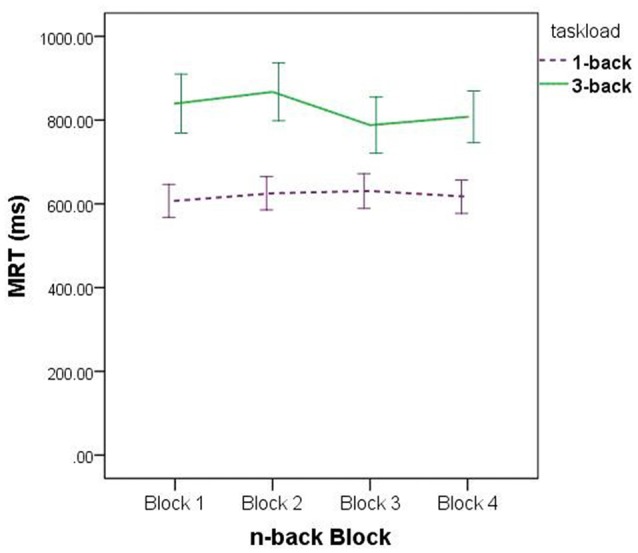
Mean Reaction Time (MRT), for all participants, on the sets of 1-back and 3-back trials.

Regarding the Task Load × Block interaction, the data depicted in Figure 3 suggest this is a product of sampling error: for the set of 3-back blocks, MRT was at its lowest on block 3, whereas for the set of 1-back blocks, MRT was at its highest on block 3. There is no apparent experimental precedent or theoretically justifiable reason for this data pattern, and hence we make no further comment about it.

In summary, we conclude from this set of analyses that neither sex nor variation in cortisol detectably affected the speed of *n*-back performance. A series of bivariate correlational analyses, documenting associations between magnitude of cortisol change from Cort_B_ to Cort_1_ and the MRT variables, confirmed the veracity of this fact, *r*_S_ < 0.40, *p*_S_ > 0.073.

### Working Memory Tasks: Secondary Analyses

The fact that our analyses detected no significant relations among cortisol, sex and WM performance did not confirm our hypotheses that cortisol elevations enhance WM performance in men, but impair WM performance in women. The results are also inconsistent with data patterns described elsewhere (e.g., Schoofs et al., 2008, 2013; Cornelisse et al., 2011). Hence, we conducted two additional sets of analyses, taking different approaches to the data and thereby assessing the fidelity of our initial findings.

The first set of analyses retained the participants’ original group assignments, and so permitted us to investigate the effects of TSST exposure (rather than elevated cortisol) on WM performance. These analyses, then, are more similar than those above to those conducted by, for instance, Schoofs et al. (2008, 2013) and Cornelisse et al. (2011). So, using the original group assignments and the log-transformed WM data, we conducted two (one on the CIS data and one on the MRT data) 4 × 2 × 4 (Experimental Condition [TSST-Women, TSST-Men, Relax-Women, Relax-Men] × Task Load [1-back, 3-back] × Block [1, 2, 3, 4]) repeated-measures ANOVAs. The only difference between these analyses and those reported above, sections “CIS” and “MRT”, is that presence of the Experimental Condition factor in place of the Group factor (i.e., the Task Load and Block data were the same as analyzed above).

These secondary analyses detected the same main effects of Task Load and of Block on CIS and MRT scores, and the same Task Load × Block interaction effect on MRT scores as did the original analysis. Most pertinent, of course is that neither secondary analysis detected main effects of Experimental Condition, *F*_S_ < 0.64, *p*_S_ > 0.59, $\eta_{\text{pS}}^{2}$ < 0.04, and that neither detected interactions involving Experimental Condition, *F*_S_ < 5.24, *p*_S_ > 0.85, $\eta_{\text{pS}}^{2}$ < 0.10. Hence, both secondary analyses detected no relations among the participant’s sex, the experimental condition to which s/he was exposed, the interaction between the two, or accuracy/speed of *n*-back performance.

Second, we created two separate general linear models to test if sex, magnitude of cortisol change from baseline to CORT_1_, and/or the interactions among those variables, account for a significant proportion of the variance in (a) CIS across the set of 3-back trials, or (b) MRT across the set of four 3-back blocks. Neither model detected main or interaction effects: for CIS, *F*_S_ < 0.78, *p*_S_ > 0.380, $\eta_{\text{pS}}^{2}$ < 0.02, *R*^2^ = 0.01, and for MRT, *F*_S_ < 1.94, *p*_S_ > 0.165, $\eta_{\text{pS}}^{2}$ < 0.03, *R*^2^ = 0.04. Again, these analyses detected no relations among sex, magnitude of cortisol change from baseline to CORT_1_, their interaction and accuracy/speed of 3-back performance.

## Discussion

A highly controlled design, featuring a modified version of the TSST and using 76 healthy adults (38 women in the late luteal phase of their menstrual cycle, and 38 men), tested the following predictions: (a) overall WM performance, as measured by the *n*-back, is faster and more accurate on a 1-back than on a 3-back task, regardless of biological sex or elevated circulating cortisol; (b) cortisol elevations enhance performance on a 3-back task in men; but (c) impair performance on a 3-back task in women.

The literature commonly reports considerable variability in response to laboratory-based stress induction methods (e.g., Buchanan and Tranel, 2008; van den Bos et al., 2009; Zandara et al., 2016). Our findings are similar: 11 participants who completed the study procedures did not respond to the TSST with measurable increases in cortisol levels. Moreover, and again as is common in this area, more women (*n* = 8) than men (*n* = 3) did not respond to the TSST with such increases (e.g., Elzinga and Roelofs, 2005; Stephens et al., 2016).

Confirming the first hypothesis, the present analyses demonstrated that, overall, participants completed the 1-back trials (presumably, representing a lighter cognitive load) more quickly and accurately than the 3-back trials (presumably, representing a heavier cognitive load). This data pattern characterized mean performance in both men and women, regardless of whether exposure to the experimental manipulation elevated cortisol. This pattern replicates data showing that people are faster and more accurate on lighter- than heavier-load WM tasks, regardless of biological sex or cortisol status (Callicott et al., 1999; Speck et al., 2000; Pelegrina et al., 2015; Rac-Lubashevsky and Kessler, 2016). This performance difference likely arises because 1-back tasks, unlike 2-back and higher tasks, require no content manipulation, are not vulnerable to interference and do not require accessing information outside of immediate attentional focus (Verhaeghen and Basak, 2005; Oberauer, 2006; Schleepen and Jonkman, 2010).

The present analyses did not confirm the second and third hypotheses, detecting no between-group differences in 3-back task performance. Hence, elevated cortisol levels did not enhance performance in men, or impair performance in women, on a heavier-load WM task.

This data pattern does not systematically replicate those described elsewhere. For example, Cornelisse et al. (2011) reported that TSST exposure facilitated faster reaction time on a 2-back (but not a 3-back) task in men. Schoofs et al. (2013), using only a 2-back task, replicated that result and found that stress exposure was associated with slower reaction times in women. Our findings are, however, consistent with those described in other studies reporting that stress-induced cortisol elevations do not affect WM performance in men (Kuhlmann et al., 2005; Oei et al., 2009; Weerda et al., 2010; Stauble et al., 2013) or women (Cornelisse et al., 2011; Zandara et al., 2016).

### On Failures to Systematically Replicate

These failures to systematically replicate data patterns under apparently similar conditions may be due to a number of between-study methodological differences including, but not limited to, the task used to represent working memory (e.g., *n*-back vs. digit span); the characterization of light and heavy cognitive loads (e.g., whereas some use 1-back vs. 2-back, others use 2-back vs. 3-back, or variations of the Sternberg WM paradigm); the outcome measures reported (e.g., accuracy vs. reaction time); the extent of practice on the tasks representing working memory (e.g., whereas some give a 0-back task and a series of practice trials at the beginning of each *n*-back block, others give two practice blocks preceding 2- or 3-back testing); if tasks representing working memory occur in isolation or as part of a task battery; the procedures used to elevate cortisol levels (e.g., hydrocortisone vs. psychosocial manipulations); the use of manipulation checks to exclude cortisol non-responders from at least some analyses; the stage of female participants’ menstrual cycle (e.g., whereas some include only female participants in the luteal phase, others include all women and do not use phase of cycle as a covariate in analyses); or if women using oral contraceptives are permitted to participate or not (Elzinga and Roelofs, 2005; Kuhlmann et al., 2005; Schoofs et al., 2008, 2013; Luethi et al., 2009; Oei et al., 2009; Weerda et al., 2010; Cornelisse et al., 2011; see Brown et al., 2014; for a detailed discussion and checklist).

In the present study, we considered the methodological choices that allowed us to best answer our questions of interest. So, for instance, we chose to use an *n*-back task because it appears to represent core WM processes more accurately than, for instance, digit span backwards. At the theoretical level, the *n*-back requires manipulation and organization of information, especially at heavier task loads (Kirchner, 1958; Veltman et al., 2003). At the level of experimental control, the *n*-back offers the option of including both accuracy and reaction time measures and can be of longer duration than digit span, where each difficulty level is typically tested with only two trials (Jarrold and Towse, 2006; Lynn and Irwing, 2008; Schoofs et al., 2008). Finally, at the empirical level, imaging studies indicate that *n*-back tasks activate the PFC reliably, and that this activation increases as cognitive load increases (Speck et al., 2000; Owen et al., 2005; Tomasi et al., 2007). Hence, even though some, based on tradition (Kane et al., 2007), or authority (Miller, 1956), justify digit span as a gold-standard measure of WM, performance on that task does not necessarily reflect the characteristics of WM as well as the *n*-back task does.

We also chose to control, by design rather than statistically, factors related to female hormonal states. Specifically, we excluded women using oral contraceptives, and we tested naturally-cycling women only in the late luteal phase of the menstrual cycle. As noted earlier, stressor-induced salivary cortisol levels obtained from men and from women in the late luteal phase are similar. Moreover, compared to men and to women in other phases of the cycle, women using oral contraception or who are in the follicular phase exhibit smaller stressor-induced cortisol increases (Kirschbaum et al., 1995, 1999).

Finally regarding methodological choices, we reconstituted our participant groups into those with and those without cortisol elevations. Doing so allowed us to focus exclusively on the major question of interest (i.e., relations between elevated cortisol and WM performance), and to maximize statistical power to detect differences relating to cortisol levels, rather than to stress exposure *per se*.

Regardless of our group reconstitution, the present pattern of cortisol data differs markedly from those reported in many other studies. The average baseline cortisol level in our sample was 1.92 (± 2.20) nmol/l. Post-manipulation, the average cortisol level in the EC groups was 5.65 (± 4.14) nmol/l. These numbers are notably smaller than those reported by most other studies using the same stress-induction method (Liu et al., 2017). Typically, studies in this field report baseline levels of over 5 nmol/l (at least one reported levels above 20 nmol/l), and post-TSST peak levels of over 10 nmol/l (at least one reported levels above 50 nmol/l; see, e.g., Kirschbaum et al., 1996; Wolf et al., 2001; Domes et al., 2004; Elzinga and Roelofs, 2005; Kuhlmann et al., 2005; Oei et al., 2006; Nater et al., 2007; Schoofs et al., 2008, 2013; Luethi et al., 2009; Cornelisse et al., 2011; Zandara et al., 2016). In the present study, the average increase in cortisol post-manipulation for the EC groups was 4.10 (± 3.47) nmol/l, a figure at the lower end of the range of increases reported by other studies, some of which describe average values of over 9 nmol/l (Kirschbaum et al., 1996; Wolf et al., 2001; Domes et al., 2004; Oei et al., 2006, 2009; Nater et al., 2007; Luethi et al., 2009; Cornelisse et al., 2011; Schoofs et al., 2013; Zandara et al., 2016). Indeed, based on the classification criteria suggested by Miller et al. (2013), 25% of participants in our EC groups (9 of 36) were “cortisol non-responders.”

The source(s) of differences between the pattern of cortisol data described in the present study and those described in some previously published studies cannot be easily explained. As in most other studies, our participants were undergraduate volunteers and we enforced standard exclusion criteria strictly. We were faithful to the TSST methodology, and an accredited and experienced laboratory staff analyzed our saliva samples (Pillay et al., 2008). Furthermore, previously published studies (Human et al., 2013; du Plooy et al., 2014) and unpublished theses and dissertations using similar samples consistently describe similar trends toward relatively low baseline cortisol levels and relatively small magnitudes of cortisol response.

We therefore propose a set of testable hypotheses, the core of which is this: “These between-study cortisol differences can be traced to the fact that our samples are drawn from the southern African population, a more racially, ethnically, culturally and genetically heterogeneous population than those from which Western European and North American laboratories sample” (Manica et al., 2007). For instance, the final sample in the present study comprised 32 white, 22 black African, and 22 colored (mixed ancestry) or Indian/Asian individuals (we use these racial terms following the descriptive nomenclature present in South African census publications; Statistics South Africa, 2015). Although previously published studies do not provide detailed racial/ethnic classification data, it is improbable that their samples were as diverse as ours.

The relatively small literature on individual racial/ethnic/cultural differences in baseline cortisol, and in magnitude of cortisol response to psychosocial stressors, may shed light on the observed discrepancies between our physiological data and those reported elsewhere. Chong et al. (2008), for example, found that, after TSST exposure, white American participants showed a significantly greater increase in cortisol levels than African-American participants. Other non-experimental studies conducted in North America report that black children have both lower basal cortisol levels and flatter cortisol curves than white children. Importantly, differences in socioeconomic status (SES) appear to strongly moderate these group differences, with black participants in those studies typically sampled from lower-SES neighborhoods or families (Chen and Paterson, 2006; Desantis et al., 2007; Dulin-Keita et al., 2012; but see Lupien et al., 2001). Although none of those studies were conducted in low- or middle-income countries (LAMICs), it is possible that low-SES individuals in LAMICs (a characterization that fits many of the participants in our sample) show diurnal cortisol rhythms (i.e., lower basal levels and flatter curves) similar to those observed in low-SES individuals in high-income countries (HICs) of North America and western Europe.

In addition, it is plausible that high-SES individuals in LAMICs have greater exposure to prenatal stress, lifetime stress and racial discrimination than high-SES individuals in HICs. Each of these adverse experiences bear a relation to both basal cortisol levels and magnitude of cortisol response to psychosocial stressors (Williams et al., 2008, 2012; Jorm and Ryan, 2014).

Furthermore, the ancestral life history (LH) strategies of individuals residing in LAMICS may differ from those residing in HICs. LH theory is an evolutionary biological framework that accounts for individual differences in reproductive strategies, suggesting that organisms inhabiting unstable environments with high mortality rates tend to produce many offspring and invest little in them (i.e., adopt faster LH strategies; see, e.g., Promislow and Harvey, 1990). Applied to humans, LH theory has guided studies seeking to understand individual differences in, for instance, the onset of puberty, age of first sexual experience and number of sexual partners (see, e.g., Belsky et al., 1991; Patch and Figueredo, 2017). Recent studies suggest cross-cultural and cross-national variations in LH strategy are associated with, for instance, variations in: (a) the parenting strategies to which individuals are exposed (Sotomayor-Peterson et al., 2012), and (b) the national prevalence of polymorphisms in the androgen receptor gene AR, the dopamine receptor gene DRD4, and the 5-HTTLPR VNTR of the serotonin transporter gene (Minkov and Bond, 2015). Of particular relevance here is that the latter study showed that nations with higher levels of socioeconomic inequality (as measured by the Gini coefficient) contained more individuals with those genetic polymorphisms, which are associated with a greater propensity toward risk-taking, dysfunctional impulsivity and unrestricted sociosexual attitudes (i.e., traits that covary with faster LH strategies). According to the World Bank (2017), South Africa has a Gini coefficient of 0.63, making it one of the most economically unequal countries in the world.

Animal studies demonstrate that variations in LH strategies are related to variations in glucocorticoid concentrations and in the strength of the HPA axis-driven physiological stress response. Although some evidence suggests that a faster LH strategy is present when basal hormone levels are lower and when stress responsiveness is dampened, there remains some debate about the precise direction and nature of this relationship (Crespi et al., 2013; Crossin et al., 2016). No published human studies address this question.

In summary, we suggest that the racial, socioeconomic and other variability we observe in our laboratory’s samples serve as proxies for long-standing cultural differences that have produced remarkable inter-individual (and perhaps genetically expressed) differences in human responses to environmental challenges (Cohen et al., 1996; Sapolsky, 2017). We could disconfirm these and related hypotheses by using a quasi-experimental design that: (a) assigns individuals to groups based on factors such as measured region of origin, individual and country SES, exposure to prenatal and lifetime stress, exposure to instances of racial/ethnic/cultural discrimination, fast vs. slow LH strategy and other variables hypothesized to be critical, and then (b) examines trends toward relatively low baseline cortisol levels and relatively small magnitudes of cortisol response in specified groups.

We have highlighted potential cultural, socioeconomic and ancestral life-history differences between participants in our study and those in previously published studies. We speculate that these differences contribute to the observed between-study differences in patterns of cortisol secretion (Oberlander et al., 2008; Huynh et al., 2016; Lovallo et al., 2017), and explain, at least partially, why the outcomes we present here are inconsistent with those obtained from less heterogeneous samples (e.g., Cornelisse et al., 2011; Schoofs et al., 2013). Specifically, if basal cortisol levels in our participants are relatively low, and the magnitude of cortisol response is also relatively small, then cortisol levels are unlikely to elevate to the supraphysiological levels known to affect cognitive performance significantly (Henry et al., 2014; Schultebraucks et al., 2015).

### Limitations

Although the control procedure we used is similar to that described by others (e.g., Elzinga and Roelofs, 2005; Cornelisse et al., 2011), it did not require participants to complete cognitive and physical tasks formally equivalent to those completed by participants in the TSST condition. The literature describes other, perhaps more intuitively appealing, control procedures (e.g., Het et al., 2009; Wiemers et al., 2013). There are, however, no empirical demonstrations or accepted standards providing guidance in these matters (i.e., there are no direct comparisons of different types of control procedures in studies with aims similar to ours).

Unlike several other studies in this literature (e.g., Cornelisse et al., 2011; Schoofs et al., 2013), we did not collect biomarkers of the sympathetic nervous system’s response to the TSST (e.g., salivary alpha-amylase; Petrakova et al., 2015). However, our focus here was on possible sequelae of cortisol elevation, rather than on those of an overall stress response (i.e., both sympathetic and HPA-axis activation), on WM performance. Hence, the collection and analysis of additional biomarker data was superfluous. Moreover, we could not use statistical analyses more sensitive to detecting sex differences in cortisol response to the psychosocial stressor (e.g., growth curve modeling; Lopez-Duran et al., 2014) because post-manipulation cortisol sampling was not dense enough. Such analyses require samples at 5–10-min intervals in the hour immediately following stressor offset.

Finally, our group of participants was too small to run meaningful analyses on cortisol samples drawn from individuals with different cultural, racial, ethnic and/or SES backgrounds. Hence, we could not give proper statistical consideration to relations among, for instance, adverse life experiences, LH strategies, cortisol responses and WM performance. As noted above, such consideration is important when analyzing data from studies in this research area, particularly if those studies are conducted using samples drawn from LAMICs in the global south.

## Summary and Conclusion

Our data analyses detected no effects of sex or cortisol variation on WM task performance. This result is inconsistent with previous reports examining sex differences, effects of stress and WM performance. However, the finding that baseline cortisol levels and magnitude of cortisol increases in our sample are substantially lower than those of other studies in the stress and cognition literature raises questions about: (a) relations among environment and physiological responses to stressors, and (b) inter-individual differences in relations between stress and cognitive performance. These questions require further investigation, especially within culturally, ethnically and socioeconomically diverse populations such as those in South Africa.

## Author Contributions

RH conceptualized and designed the study, collected and analyzed data, wrote the first draft of the manuscript and was involved in re-writing and editing the final version of the manuscript. MH assisted in conceptualizing and designing the study, collected data and was involved in re-writing and editing the final version of the manuscript. WJJ was involved in re-writing and editing the final version of the manuscript. KT assisted in conceptualizing and designing the study, and was involved in re-writing and editing the final version of the manuscript.

## Conflict of Interest Statement

The authors declare that the research was conducted in the absence of any commercial or financial relationships that could be construed as a potential conflict of interest.
